# An Outbreak of Community-Acquired Foodborne Illness Caused by Methicillin-Resistant *Staphylococcus aureus*

**DOI:** 10.3201/eid0801.010174

**Published:** 2002-01

**Authors:** Timothy F. Jones, Molly E. Kellum, Susan S. Porter, Michael Bell, William Schaffner

**Affiliations:** * Tennessee Department of Health, Nashville, Tennessee, USA; † Centers for Disease Control and Prevention, Atlanta, Georgia, USA; ‡ West Tennessee Regional Health Department, Jackson, Tennessee, USA; § Vanderbilt University School of Medicine, Nashville, Tennessee, USA

**Keywords:** foodborne, community-acquired, methicillin resistant Staphylococcus aureus

## Abstract

Infections with methicillin-resistant *Staphylococcus aureus* (MRSA) are increasingly community acquired. We investigated an outbreak in which a food handler, food specimen, and three ill patrons were culture positive for the same toxin-producing strain of MRSA. This is the first report of an outbreak of gastrointestinal illness caused by community-acquired MRSA.

Infection with methicillin-resistant *Staphylococcus aureus* (MRSA) has been reported in the United States for over 30 years. Initially, MRSA infections were primarily a problem of hospitals and nursing homes; by 1997, 50% of health-care-acquired *S. aureus* isolates in the United States were methicillin resistant (*1*). Beginning in the early 1980s, cases of community-acquired MRSA were reported, primarily in persons with a history of injection drug use and other high-risk patients (*2*). More recently, community-acquired MRSA has been described in both adults and children who did not have extensive exposure to hospitals or other apparent risk factors (*3*,*4*). We describe the first report of a community-acquired outbreak of acute gastroenteritis caused by MRSA.

## Outbreak Report

A family purchased shredded pork barbeque and coleslaw from a convenience-market delicatessen. The pork was reheated in a home microwave, and three adults ate the food 30 minutes after it was purchased. Approximately 3 to 4 hours after eating the meal, the three adults--who had not eaten another common meal together in the preceding week--had nausea, vomiting, and stomach cramps. Two children at the dinner who did not eat barbeque or coleslaw did not become ill. Two of the three ill adults were taken to a hospital for evaluation, where they were treated and released. Vomiting ceased after treatment with phenothiazine, and nausea and cramps resolved the following day.

## Methods

Ill family members were interviewed by the local health department, and an environmental inspection was performed at the market where the food was purchased. Market employees were interviewed, and stool cultures were obtained from the three ill persons. Specimens of barbequed pork and coleslaw were collected from the market, and nasopharyngeal swabs were collected for culture from three food preparers. Follow-up nasopharyngeal cultures were obtained from one ill family member 8 months after her acute illness to assess persistent carriage.

Twelve cultures of *S. aureus* recovered from stool samples of the ill family members, food specimens, and nasal swabs of the food preparers were sent to the Centers for Disease Control and Prevention for further testing. The identification of all *S. aureus* isolates was confirmed by conventional biochemical tests, and all isolates were screened for methicillin resistance by using a 1-µg oxacillin disk. Five of the 12 isolates appeared to be methicillin resistant by disk diffusion. Oxacillin susceptibility of the isolates was confirmed by broth microdilution (*5*) with plates read manually. All isolates were tested for staphylococcal enterotoxins by the use of a reversed passive latex agglutination test (Oxoid Ltd., Hampshire, UK). Molecular typing of all isolates was performed by pulsed-field gel electrophoresis (PFGE) with Sma I-digested chromosomal DNA. Gels were interpreted by standard criteria (*6*).

## Results

*S. aureus* was recovered from the stool cultures of the three ill persons, three samples taken from the barbequed pork, one sample from the coleslaw, and five nasal swabs from three food handlers at the convenience market. Comparison of all the isolates by PFGE showed that five isolates were indistinguishable (Figure). These isolates were those from the stool cultures of three family members (A, B, and C); the coleslaw; and from the nasal swab of food preparer C. This strain produced staphylococcal enterotoxin C and was identified as being MRSA. These isolates were resistant to penicillin and oxacillin but sensitive to all other antibiotics tested. Two different strains of *S. aureus* recovered from the nasal swab of food preparer B also produced staphylococcal enterotoxin C and differed only slightly from the MRSA strain by PFGE. However, both isolates were methicillin-sensitive *S. aureus* (MSSA). These two isolates were categorized as closely related subtypes of the outbreak strain. The *S. aureus* isolate recovered from the third food preparer (A) was MSSA, produced staphylococcal enterotoxin A, and was determined by PFGE to be unrelated to the outbreak strain. Although the *S. aureus* isolates from the samples of pork barbeque each produced staphylococcal enterotoxin C, they were MSSA and unrelated to the outbreak strain by PFGE.

**Figure:**
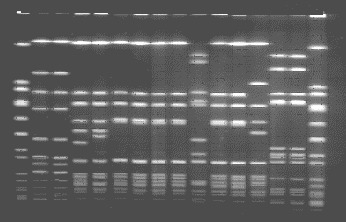
Pulsed-field gel electrophoresis profiles of Sma I-digested chromosomal DNA. Lanes 1 and 16, NCTC 8325 standard; lane 2 and 3, methicillin-sensitive *Staphylococcus aureus* (MSSA) nasal isolates from food preparer A; lanes 4 and 5, MSSA nasal isolates from food preparer B; lane 6, methicillin-resistant *S. aureus* nasal isolate from food preparer C; lane 7, MRSA stool isolate from family member A; lane 8, MRSA stool isolate from family member B; lane 9, MRSA stool isolate from family member C; lanes 10 and 11, MSSA follow-up isolates from family member C; lane 12, MRSA isolate from slaw; lanes 13, 14, and 15, MSSA isolates from barbequed pork.

The barbequed pork and coleslaw were prepared at the store where they were purchased. An environmental inspection of the facility performed after the outbreak revealed no apparent lapses in technique or procedure that would have contributed to the outbreak. No additional cases of illness related to this outbreak were reported to the local health department.

Food handler C, who was carrying the outbreak strain of MRSA, performed various tasks at the store, including preparing foods and handling barbecued pork and coleslaw. She reported no recent gastrointestinal illness nor chronic health problems, history of admission to a hospital, or use of antibiotics in the previous 6 months. She also denied close contact with persons who lived or worked in health-care facilities or other group settings. She did, however, visit an elderly relative, who resided in a nursing home, approximately 2 to 3 times each month before the outbreak. She reported that this person had a staphylococcal infection and had subsequently died. The employee refused to identify her relative, and further medical information or isolates from that person were not available.

A follow-up nasopharyngeal culture was collected from family member C approximately 8 months after her acute illness. This culture was positive for two different strains of MSSA, but not MRSA. One isolate was indistinguishable by PFGE from that of the MRSA strain isolated from the same patient during the outbreak. This isolate produced enterotoxin C, as did the strain of MRSA she was previously carrying. Polymerase chain reaction testing of this isolate confirmed that it carried the mecA gene, suggesting that the original MRSA strain had reverted to MSSA by loss of a regulatory region. The other isolate, which produced enterotoxin D, was determined by PFGE to be unrelated to any of the other strains previously seen in this investigation.

## Conclusions

Despite its ubiquity as a health-care-acquired pathogen and increasing reports of community-acquired infections, MRSA has not previously been reported as a cause of outbreaks of gastroenteritis. Staphylococcal food poisoning is estimated to account for 185,000 foodborne illnesses per year in the United States; most of these go unreported (*7*). Because staphylococcal food poisoning is toxin mediated and generally self-limited, antibiotics are not used for therapy. Also, many *S. aureus* isolates obtained as part of outbreak investigations may not be tested for antibiotic susceptibility, and therefore methicillin-resistant strains may go unrecognized as the cause of foodborne outbreaks of acute gastroenteritis. Methicillin-resistant strains of *S. aureus* are as likely to produce enterotoxins as are methicillin-sensitive strains. Therefore, outbreaks of acute gastroenteritis due to MRSA are not unexpected.

Until recently, MRSA has been considered primarily a health- care-associated pathogen, causing invasive disease in which multidrug resistance poses a substantial challenge to successful treatment. Food has been implicated as a source of spread in one outbreak of blood and wound infections in hospitalized immunocompromised patients (*8*). There has been some debate about the appropriateness of the term “community-acquired” to describe many *S. aureus* infections in which distant hospital exposures cannot be excluded with certainty and colonization can persist for years. In this outbreak, it appears that MRSA-contaminated food was the vehicle in a cluster of illnesses affecting low-risk persons within the community and that this food was likely contaminated by an asymptomatic carrier whose only apparent exposures were intermittent visits to a nursing home. This outbreak could be an example of second-generation spread of a health-care-associated pathogen into the community. The outbreak strain of MRSA, however, was resistant only to penicillin and oxacillin and was sensitive to all other antibiotics tested. A strain originating in a health-care facility would have an increased likelihood of being multidrug resistant (*9*).

MRSA was isolated from a food handler involved in this outbreak, and an MSSA strain with an identical PFGE pattern was subsequently isolated. The strain obtained on follow-up retained the mec-A gene and probably represents a genetic mutation in a regulatory region. Shortly after the outbreak, another food handler was carrying two strains of MSSA with a PFGE pattern very similar to the outbreak strain of MRSA. These strains appear to be related to the outbreak strain but with loss of the mec-A determinant. The existence of multiple strains of *S. aureus* in persons involved in this outbreak is not surprising, as 20% to 40% of adults are estimated to be colonized at any time, and multiple strains may be present in the same person. Colonization with MRSA carries a greater risk for infection than does colonization with MSSA (*10*).

This outbreak suggests that as MRSA becomes increasingly common in the community, it will be implicated in expressions of all the clinical manifestations of staphylococcal infection. While antibiotic-resistant strains are not expected to be clinically more virulent or challenging in the setting of acute outbreaks of gastroenteritis, MRSA may cause soft-tissue and other infections in the community that are difficult to treat. This episode demonstrates the spread of MRSA into the community and is a harbinger of the increasing impact of health-care-associated organisms in settings and among populations previously considered to be unthreatened by this emerging antimicrobial-resistant pathogen.
